# Roles of Microglial Ion Channel in Neurodegenerative Diseases

**DOI:** 10.3390/jcm10061239

**Published:** 2021-03-17

**Authors:** Alexandru Cojocaru, Emilia Burada, Adrian-Tudor Bălșeanu, Alexandru-Florian Deftu, Bogdan Cătălin, Aurel Popa-Wagner, Eugen Osiac

**Affiliations:** 1 Department of Physiology, University of Medicine and Pharmacy of Craiova, 200349 Craiova, Romania; cojo.alexandru92@gmail.com (A.C.); emilia.burada@umfcv.ro (E.B.); alex.deftu@bio.unibuc.ro (A.-T.B.); 2 Experimental Research Center for Normal and Pathological Aging, University of Medicine and Pharmacy of Craiova, 200349 Craiova, Romania; 3 Pain Center, Department of Anesthesiology, Lausanne University Hospital (CHUV), CH-1011 Lausanne, Switzerland; adrian.balseanu@umfcv.ro; 4 Faculty of Biology and Medicine (FBM), University of Lausanne (UNIL), CH-1011 Lausanne, Switzerland; 5 Chair of Vascular Neurology, Dementia and Ageing Research, University Hospital Essen, 45147 Essen, Germany; 6 Department of Biophysics, University of Medicine and Pharmacy of Craiova, 200349 Craiova, Romania; eugen.osiac@umfcv.ro

**Keywords:** microglia, ion channel, neurodegeneration, cerebral ischemia, alzheimer’s disease, parkinson disease, multiple sclerosis, Lateral amyloid sclerosis, epilepsy

## Abstract

As the average age and life expectancy increases, the incidence of both acute and chronic central nervous system (CNS) pathologies will increase. Understanding mechanisms underlying neuroinflammation as the common feature of any neurodegenerative pathology, we can exploit the pharmacology of cell specific ion channels to improve the outcome of many CNS diseases. As the main cellular player of neuroinflammation, microglia play a central role in this process. Although microglia are considered non-excitable cells, they express a variety of ion channels under both physiological and pathological conditions that seem to be involved in a plethora of cellular processes. Here, we discuss the impact of modulating microglia voltage-gated, potential transient receptor, chloride and proton channels on microglial proliferation, migration, and phagocytosis in neurodegenerative diseases.

## 1. Introduction

Neurological diseases are a major cause of death and disability worldwide [1]. Within the plethora of neurological diseases, a particular subcategory of pathologies are neurodegenerative diseases. In the most classical view, neurodegenerative diseases are the result of a slow neuronal loss. Alzheimer’s (AD) and Parkinson’s disease (PD) are the prototypical neurodegenerative diseases. However, neuronal loss can also occur in other pathologies that are not typically found in textbooks of neurology. Such pathologies follow acute events such as stroke or traumatic brain injury, where in the weeks and even years after the initial event, a cascade of neuronal loss is initiated [2,3]. Regardless of the primary cause of neurodegeneration, it will always involve neuroinflammation. This will lead to a so-called microglia activation in which the morphology the cells change dramatically [4]. This is because microglia are considered the first responders within the central nervous system (CNS) [5]. When activated, microglia show an increase in basic cellular functions such as proliferation, migration, cytokine secretion and phagocytosis [6,7,8]. Therefore, microglia morphology is directly linked to its intracellular activity.

Microglia morphology varies according to the phenotype expressed in response to certain stimuli and to the environment: it has a surveillance function with a high number of processes from which it can switch to an amoeboid form that is characterized by the presence of multiple intracellular vesicles with a role in phagocytosis [9]. The general consent is that there are two phenotypes, M1 and M2 which are present in various neurological disorders. M1 is considered a pro-inflammatory state and M2 denotes an anti-inflammatory state. The classic activation to the pro-inflammatory (M1) state is determined by IFN-γ and is associated with the production of TNF-α, IL-1b and nitric oxide (NO) [9,10]. The alternative anti-inflammatory state occurs in response to IL-4, IL-13, and arginase [11].

Microglia can have either a beneficial or a harmful role in neurodegenerative diseases. There are several studies claiming that the inflammatory process plays an important role in the disease progression by facilitating amyloid beta (Aβ) deposition, neuronal damage and cognitive deficits [12,13]. Animal models of AD with cerebral Aβ accumulation show an increased expression of pro-inflammatory chemokines and cytokines, such as TNF-α, IFN-γ, IL-1b, and IL-6 [14,15,16], and there is evidence showing that the anti-inflammatory profile of microglia has an important role in reducing Aβ accumulation in AD [17].

**Figure 1:**
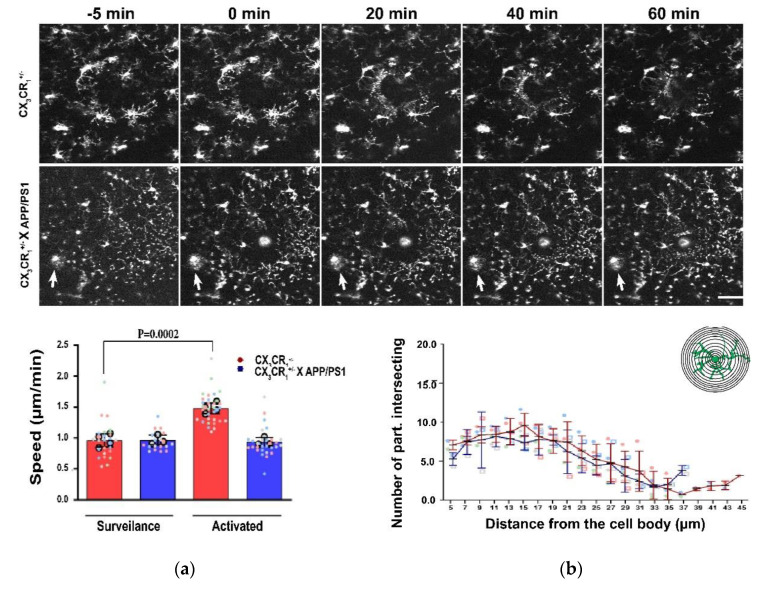
Time-lapse recordings of the same cortical region before and after a micro lesion was done with a laser shows how microglia will rapidly isolate the lesion site from the healthy brain, as to stop the spread of the damage. (**a**) In the presence of amyloid microglia are slower to react to the lesion although the surveillance speed in the intact parenchyma is comparable between tissue with and without amyloid; (**b**) Analyzing microglia morphology using a Scholl approach has revealed no difference in the number of intersections from the cell body but revealed that in the presence of amyloid, microglia appear to have a shorter process arborization. Data collected from 4 animals only expressing CX_3_CR_1_ (29 analyzes cells) and from 3 animals expressing both CX_3_CR_1_ and APP/PS1 mutations (26 analyzed cells). Mean and standard deviation is shown. APP: amyloid precursor proteins; PS1: presenilin 1.

There are numerous experimental studies, including unpublished observations from our own group (Figure 1), that support changes in microglia behavior and morphology [18,19] in response to Aβ accumulation. Of note, even nanomolar concentrations of Aβ oligomers can activate microglia, via the potassium KCa3.1 channel [20]. As such, it does not seem so farfetched that microglia morphology could be an early indicator of AD.

Despite all the data related to the genetic involvement in AD patients under 65 years of age, less than 10% of the cases were found to be caused by a mutation in the genes coding for amyloid precursor proteins (APP), presenilin 1 (PSEN1) and/or presenilin 2 (PSEN2) [21]. Although several single genetic factors have been linked to the onset of late AD, it is now known that AD is a multifactorial disease [22]. Further, comorbidities such as diabetes, high blood pressure, obesity, dyslipidemia and other cerebrovascular disease have been shown to increase the risk of developing AD [23].

Since microglia ion channels are key regulators of microglial function and morphology, in this review, we aim to analyze their roles in cerebrovascular diseases and discuss how their modulation can be used for therapeutic purposes.

## 2. Microglia Potassium Channels

Microglia are found in all brain structures but in some regions their density is higher [24]. In AD, numerous activated microglia can be found in the hippocampus which is the most affected region of the brain [25]. A study conducted on isolated microglia, showed that only Kv1.3 channels have a direct role in cell proliferation. Of note, Kv1.5 channels are not involved in this process [26]. On the contrary, it seems that Kv1.5 channels predominate in surveillance microglia (M2 phenotype) while activated microglia switch to Kv1.3 channels [27]. However, since potassium channels have multiple functions and are found in all cells of the human body, these observations may be just the tip of the iceberg. These channels activation causes cell membrane hyperpolarization which in turn lead to calcium influx by activating calcium channels [28,29].

Various types of potassium channels in mice microglia were identified by different molecular techniques: voltage-gated potassium channels (Kv1.3, Kv1.1, Kv1.5, Kv3.1) [30] and calcium-activated potassium channels [22]. It has also been observed the presence of some particular potassium channels, such as Kv4.1 and K2P13.1 both in mice with experimentally induced autoimmune encephalomyelitis, and in human microglia cultures [31]. Microglia potassium channels have been reported to have a high heterogeneity in different species, displaying a variety of expression patterns according to microglia activation mode, M1 or M2 [32].

Neuroinflammation has been linked to microglia potassium channel changes that impact on cellular behavior [33]. Numerous studies conducted in animal models, especially in rodents, highlight the existence of potassium channels variability according to the rodent model [34]. For example, Kv1.3 has been involved in BV2 microglia migration [35] while Kir2.1 has been implicated in resting membrane potential of the spinal cord microglia [33]. After lipopolysaccharides (LPS) activation of microglia in the neonatal mice CNS, there was an increase in Kv1.3 expression in microglia, with no influence on the expression of Kir2.1 and KCa3.1 [36,37].

Studies conducted on human microglia from epilepsy patients, showed a higher expression of KCa3.1 and of the inward rectifier potassium channels, Kir, no matter if cells were LPS or IL-4 stimulated [38]. What was surprising, however, is that these Kv1.3 channels were specifically immunohistochemically labeled in the microglia surrounding amyloid plagues in AD patients [39], and in cells surrounding active multiple sclerosis lesions [40] and also in microglia of stroke patients [41]. This demonstrates that different neuropathological conditions can modulate potassium channels expression in a way that we cannot yet integrate in the overall pathology. Furthermore, potassium channels expression in microglia is age-dependent [42,43]. Thus, in elderly mice microglia have a more negative resting membrane potential, as well as an increased expression of inward and outward rectifier potassium channels, as demonstrated by studies conducted both in vitro and in vivo [44].

In neuroinflammatory processes that take place in AD, voltage-gated potassium channels, especially Kv1.3 channels, have a very important role in modulating microglial activity [39]. In primary cultures, microglia increase the expression of Kv1.3 after exposure to a small amount of soluble Aβ [45]. Still, another study has shown that the Aβ1-42 fraction modulates the activation/inactivation of Kv1.3 channels, without changing the channel conductance [46]. A similar activation was observed for KCa3.1 channels even before the plaque formation after exposure to very low concentrations of beta amyloid oligomers (ABO) [20]. Furthermore, exposure to ABO increased the number of immunoreactive CD11b microglia that express high levels of KCa3.1 in the brains of 5xFAD mice [47] and AD patients. Of note, application of *senicapoc*, a KCa3.1 blocker, reduced neuroinflammation and synaptic damage induced by ABO [47]. Using the same KCa3.1 blocker in mice models, which were injected with MPTP (1-methyl-4-phenyl-1,2,3,6-tetrahydropyridine), a Parkinson’s-inducing neurotoxin, improved the locomotion, attenuated microgliosis and neuroinflammation in the substantia *nigra pars compacta* and reduced dopaminergic (DA) neuronal loss [48]. All these observations seem to be related to the specific microglia role of KCa3.1 that was linked to NO and ABO-induced microglia neurotoxicity [20]. Interestingly, the administration of a broad-spectrum potassium channel inhibitor (4-aminopyridine) was successful in inhibiting microglial activity, and reduced the neuronal damage inflicted by the M1 phenotype [49].

Aminopyridine can also block Kv1.3 potassium channels and is able to reduce microglia reactive oxygen species production [50]. Long-term use of a Kv1.3 selective blocker, 5-(4-fenoxybutoxy)psoralen (PAP-1) in APP-PS1 mice, reduced Aβ accumulation causing synaptic recovery in the hippocampus [45]. An increase in microglia Kv1.3 expression was observed after 2 days following reversible middle cerebral artery occlusion (MCAo), with a peak at 8 days after reperfusion. A dose dependent response was observed in the infarction area by blocking Kv1.3 channels using PAP-1. This effect was attributed to the modulatory effect of PAP-1 on proinflammatory cytokines levels by reducing the concentrations of IL-1β and IFN-γ, with no effect on IL-10 and brain-derived nerve growth factor (BDNF) [51]. Similarly, the treatment of mice with TRAM-34 which blocks KCa3.1 channel, resulted in a reduced stroke volume and improved neurological deficits [41,52].

## 3. Microglia Calcium Channels

There are three main types of calcium channels found at the membrane level: voltage-gated calcium channels (VGCC), receptor-operated calcium channels (ROC) and storage operating calcium channels (SOC). Voltage-gated calcium channels are responsible for calcium homeostasis maintenance, gene expression control, various cellular processes, hormone secretion and cell apoptosis [53]. These channels are divided into high voltage-activated channels, Cav1 (Cav1.1–1.4), Cav2 (Cav2.2–2.3), and low voltage-activated channels Cav3 (Cav3.1–3.3) [54]. There are numerous studies carried out on glial cells mixed cultures, including microglia, that have demonstrated the existence and functionality of voltage-gated calcium channels in these cells [47,53,55,56].

One of the earliest observations regarding Ca^2+^ implications in AD was the exposure of human microglia cultures to Aβ that resulted both in increased human microglial proliferation and increased concentration of intracellular Ca^2+^. By blocking calcium channels using verapamil, nifedipine and diltiazem, a simultaneous decrease in both intracellular calcium levels and microglial proliferation was observed. Furthermore, by removing Ca^2+^ from the medium, the accumulation of intracellular Ca^2+^ was reversed, which emphasized the role of voltage-gated calcium channels in calcium influx [57]. Recently, the involvement of Cav2.1 and Cav2.2 channels in glial cell proliferation was reported [56], emphasizing the roles of voltage-gated calcium channels have in neurotoxicity [55].

Interspecies heterogeneity and the importance of calcium channels in AD is even more impressive considering that animal studies were not able to describe the existence and activity of voltage-gated calcium channels in microglia. It is not clear if this is due the reduced or even undetectable activity of these channels in rodent’s microglia [58]. Another aspect to consider is that increased long-term intracellular level of Ca^2+^ can influence signaling in microglia, leading to microglial activation and the progression of various conditions such as Alzheimer [58]. A recent study showed that LPS and IFN-γ stimulation caused microglial activation that led to both morphological changes and L-type currents activity adjustments in animal models of neurodegeneration. Moreover, after nifedipine or Bay-K8644 (a calcium antagonist) administration, led to changes in intracellular calcium concentration, suggesting that voltage-gated calcium channels are involved in microglia activation and induction of the pro-inflammatory phenotype [59].

Several calcium blockers have been involved in anti-aging research. For example, dihydropyridine-based calcium blockers activate the anti-aging neuroprotective protein, sirtuin 1 [60], or regulate and modulate autophagy [61]. Given the involvement of calcium channels in AD, several studies have investigated the possibility of modulating Ca^2+^ channels in AD [62,63]. For example, nivaldipine (a dyhidropyridine calcium channel blocker) was reported to improve the cognitive function of AD mice [64], while isradipine, a class of dihydropyridines that block Cav1.2 subtype, was reported to prevent memory loss by targeting the expression of L-type calcium channels (LTCC) in the CA1 region of the hippocampus in a mouse model of AD [65]. Furthermore, experimental in vivo studies found that mice receiving isradipine, have a decreased deposition of Aβ1-40 and Aβ1-42, and could regulate autophagy via the LC3B protein [66]. However, some experimental data showed an increase in the Aβ1-42 secretion after the nimodipine treatment [67]. However, in clinical trials, the effect of nimodipine could not be confirmed [68]. In Cav1.2 knockdown mice, administration of MPTP resulted in intensive degeneration of dopaminergic neurons and accompanying behavioral deficits, suggesting that blocking these microglia channels in PD could have a detrimental effect [69].

Apo-E and the associated Ca^2+^ signaling have been involved in neuronal damage inflicted by stroke [70] or by Abeta accumulation in AD [71,72,73]. Further, feeding Apo-E knockout mice on a high-cholesterol diet (HCD) for 10 weeks, caused a more severe ischemic injury when compared to mice fed a normal diet in a MCAo model of stroke. Of note, administration of amlodipine, a non-selective calcium blocker to the HCD mice for 10 weeks, was able to reduce the size of the ischemic lesion, improve neurological deficiency and decrease the production of super-oxides in the perilesional area. Similar results were reported for animals that were treated for two weeks only [70].

Given the advanced age of most AD patients, it is unclear if AD itself can be a risk factor for the new SARS COV2 infection [74,75]. The effect of SARS COV2 infection on the brain will only be assessed in the long run. However, we know that viral infections can have a profound effect on VGCC, ROC, SOC, transient receptor-potential ion channels, and that Ca^2+^-ATPase disturbances cause the growth of intracellular Ca^2+^ [76,77,78]. There are no studies as yet to show the interaction between SARS COV 2 and Ca^2+^, but having a sequence homology to other coronaviruses (SARS and MERS COV) in which the link with Ca^2+^ has been demonstrated [79,80], we can assume that the new virus may be a risk factor for AD. However, although the studies on SARS and MERS COV have failed to show an aggravation of AD secondary to the infection [81], the new SARS COV2 could be a different story. 

## 4. Microglia TRP Channels

Another class of channels expressed in microglia cells that are of particular interest for neurodegeneration is represented by the potential transient receptor (TRP) channels [22,82]. TRP channels are expressed both in neuronal and other types of immune and inflammatory cells [83,84]. Among the six TRP channel subfamilies, only the TRPC (canonical), TRPV (vanilloid), TRPM (melastatin) are expressed in microglia [85,86,87]. Therefore, in this review we will focused on just the aforementioned three channel subfamilies.

TRPC are nonselective Ca^2+^ permeable cation channels that are well expressed both in neurons and glial cells [88]. The expression of TRPC seems to differ between cultured microglia where TRPC1, TRPC3 and TRPC6 are well expressed [89] and in vivo microglia where TRPC3 is poorly expressed [90]. The importance of these channels in neurodegenerative disease is taking a center stage as Ca^2+^ signaling is more and more linked both to cell death triggering and cell death inhibition [91,92]. The altered TRPC channel function in AD is still a speculation. However, theories regarding free radical production, endothelial reticulum stress and mitochondrial dysfunction all seem to imply alterations in Ca^2+^ homeostasis and TRPC channels [93].

The levels of BDNF, a known activator and upregulator of TRPC3 channels [94,95] are known to be negatively regulated during AD progression [96,97], and this is independent of BDNF polymorphisms [98]. Given the importance of TRPC3 in oxidative stress [94] and the fact that deleting the PS1 gene results in a higher intracellular concentration of Ca^2+^ [99], it is possible that these channels could be implicated in either the onset or the progression of the disease.

Inhibition of TRPC channels led to a reduction in the number of microglia and astrocytes around the inflammatory site, decreased cerebral edema and neuronal damage and improved neurological deficit after cerebral haemorrhage, thus highlighting their importance in the development of neurodegenerative diseases [100].

The increased oxidative stress in AD and subsequent mitochondrial dysfunction [101,102] seems to be linked to another TRP microglial channel from the vanilloid family: TRPV1. However, pharmacological manipulation of TRPV1 channels yielded conflicting results. For example, the administering of capsaicin, a TRPV1 channel agonist, to dorsal root ganglion neurons, caused an increased Ca^2+^ influx leading, in turn, to elevated levels of reactive oxygen species (ROS) [103]. By modulating the TRPV1 activity using capsaicin in an experimental model of PD, a positive effect was reported for survival of dopaminergic neurons in the substantia nigra. The effect was reversed when an antagonist was given [104]. Although microglia express other TRPV channels, like TRPV4 [105] and TRPV2 [106], no data of their involvement in neurodegeneration is available. However, data regarding TRPV4, for example, show a potential role in decreasing microglia activation and TNF-α production [102,107], which could theoretically, prevent excessive microglial activation and decreased neuronal apoptosis under pathological conditions [108].

Regarding TRPV2 channels, their physiological role in microglial cells is still unknown, but these channels are known to be located in the endoplasmic reticulum, where high temperature, mechanical factors and other ligands have been shown to activate them [109]. Because TRPV2 channels have a role in the intracellular signaling mechanism of Ca2^+^, they might play a role in microglial activation, too [106].

Known for their involvement in the persistence of neuropathic pain, an elevation of chemokine CXCL2 (C-X-C motif-ligand 2) and NO in microglia aggravates the pro-nociceptive response caused by inflammation [110,111]. In this context, TRPM2 channels have been recently linked to AD, as a potential molecular pathway of microglia activation following increases in Aβ-42 aggregates [112]. This is of particular interest because mice lacking TRPM2 and producing large quantities of Aβ were reported to have an improved age-related spatial memory deficit and a reduction in microglial activation in the hippocampus [113]. Although important for microglia migration and activation [114,115], the involvement of TRPM4 channels in neurodegeneration has yet to be determined.

## 5. Microglia Voltage-Gated Sodium Channels

Voltage-gated sodium channels (Nav) play an important role in all excitable cells. Nav are formed by associating alpha and beta subunits. Alpha subunits are activated by changes in the membrane potential and act as “voltage sensor” [116]. The role of beta subunits is to control channel gating and also act as cell adhesion molecules by interacting with the cytoskeleton and extracellular matrix [117].

Cellular membrane depolarization generates Na+ transient currents which initiate action potentials in myocardium, skeletal muscle and neurons. Although microglia are non-excitable cells, they express sodium channels in both physiological and pathological conditions [118,119]. In 1952, Huskin and Huexley first described sodium currents using patch clamp techniques. Since 1980, nine alpha-forming subunits of sodium channels, Nav1.1–Nav1.9, encoded by the SCN1A-SCN11A genes, have been discovered [120]. Further studies showed some channel specificity for certain cell types: neurons express Nav1.1–Nav1.3 and Nav1.6–Nav1.8 while Nav1.4 is found in myocytes and Nav1.5 is expressed by cardiomyocytes [121,122]. A great controversy over the functional role of these channels has ignited when such channels were discovered in non-excitable immune system cells, such as lymphocytes [123,124,125,126,127], macrophages [128,129], fibroblasts [130], osteoblasts [118] and other cells.

Nav in glial cells have been highlighted for the first time by blocking the fast-activating and fast-inactivating currents using specific antagonists: saxitoxin (STX) and tetrodotoxin (TTX) on astrocytes [131] and Schwann cells cultures [132]. Subsequent studies have shown the presence of these channels in oligodendrocytes [133,134] and microglia [135,136]. The patch-clamp technique has allowed to classify the alpha subunits of the sodium channels by their sensitivity to nanomolar concentrations, respectively micromolar concentrations of TTX. It has been observed that Nav1.1-Nav1.4, Nav1.6 and Nav1.7 subunits are blocked by nanomolar concentrations of (TTX-S) while Nav1.5, Nav1.8 and Nav1.9 subunits are blocked by micromolar concentrations of (TTX-R) [135,137]. Although microglia in the CNS express several voltage-gated sodium channels, Nav1.1, Nav1.5 and Nav1.6, only the latter isoform is predominantly active [137]. Thus, despite low levels of Nav1.6 in the CNS [138], highly increased levels of Nav1.6 were observed in experimental induced autoimmune encephalomyelitis (EAE) [139]. This indicates that the expression levels of microglial Nav1.6 channels is determined by the severity of the disease, and reflect the morphological transformation of microglia into the amoeboid form as demonstrated in optic nerve and spinal cord cells [139]. Another study performed on microglia cell cultures showed that cell activation using adenosine triphosphate (ATP) increases Nav1.6 expression, especially in lamellipodia [135].

Nav involvement in AD is not precisely known. However, according to a study, exposure of human microglial cells to amyloid beta did not cause significant changes in sodium flow through Nav1.5 channels [136]. This could be due to a number of factors, such as non-standardized laboratory protocols, preparation of exogenous beta amyloid or availability of various Aβ species.

Microglia in the CNS are activated under certain pathological conditions and is characterized by migration, phagocytosis, secretion of chemokines, cytokines and ROS [9,140,141]. In an EAE mouse model, the administration of phenytoin, an antiepileptic sodium channel blocker, led to a four-fold decrease in the number of microglia [142]. Using a similar rat model of EAE, a specific axonal protection was observed after safinamide (a sodium blocker) administration. Although safinamide was administered after the onset of EAE symptoms, only 10% of the treated rats developed bilateral paralysis compared to 65% in the control group treated [143] proving once more the need to better understand how ion channels can be used to modulate the inflammatory response.

By blocking sodium channels, especially Nav1.6, certain microglial functions can be modulated [137]. Nav blockade with TTX and phenytoin led to an approximately 40% reduction of phagocytosis in cultures of lipopolysaccharide-stimulated microglia (LPS) and approximately 65% reduction in the number of microglia derived from mice that do not express Nav1.6 [139,144]. Furthermore, the use of TTX and phenytoin, reduces inflammatory cytokines and chemokines release, including interleukin-1α, IL-1b and TNF-α from reactive microglia, but with minimal effects on IL-2, IL-4, IL-10, IL-6, monocyte chemotactic protein 1 (MCP-1), and transforming growth factor α (TGF-α). Also, safinamide treatment reduces superoxide production and increased synthesis of the antioxidant glutathione in activated microglial culture [143].

Another essential function of microglia is to migrate to regions of the nervous system where various pathological processes take place. The migration process is complex and very well coordinated and involves several cellular pathways such as transduction of external migratory signals, adhesion and withdrawal of the membrane, microglial polarization and rearrangement of cytoskeletal proteins [22]. The first stage of the migration process is lamellipodia formation [145], which is a membrane prominence containing actin F, actin binding proteins, Ca^2+^ and GTP binding signaling proteins, and the Rac protein [146]. One study analyzed the role of Nav in the migration process of microglia and reported a 50% reduction in the number of microglia that was activated by exposure to ATP followed by TTX or phenytoin administration [135,137].

In glia cells, Na^+^ ions play a role in maintaining both intracellular and extracellular calcium through the Na^+^/Ca^2+^ exchanger (NCX). Na^+^ is transported according to the concentration gradient, i.e., Na^+^ is introduced into cells and Ca^2+^ is exported. In the case that Na^+^ concentration is decreased or the cell is depolarized, NCX exports Na^+^ and imports Ca^2+^ in an attempt to restore the normal membrane potential [147]. In traumatic brain injuries, the flow of Na^+^ increases through voltage-gated channels and causes a reverse working mode of the NCX that leads to an increased concentration of intracellular Ca^2+^ [147]. Interestingly, GABA administration led to increased levels of intracellular Ca^2+^ and Na^+^, and consequently, to precipitous microglial migration [148]. It has been observed that administration of specific siRNAs blocks Na^+^ channels and leads to a decrease in intracellular Na^+^ and Ca^2+^, thereby reducing migration of microglia cells [148].

Blocking NCX with TTX or Nav1.5 siRNA reduces the wound healing time [149]. Given that both Rac1 (a protein with role in migration) and mitogen-activated protein kinase (MAPK), a key enzyme involved in proinflammatory processes, are modulated by Ca^2+^ concentration [150] that in turn are modulated by Na^+^ levels, blockade of voltage-gated sodium channels can inhibit microglial migration [135,137].

Nav1.6 microglial channels have a decreased expression in the normal CNS and an increased expression in neuropathologies [139]. This seems to be true for both animal models where the injection of MPTP in C57BL/6J mice generates an increase in microglia activation and an upregulation of microglial Nav1.6 channel [139,151]. This effect was reversed with zonisamide [151]. Taken together, this data shows that chronic neurodegeneration is associated with changes in sodium channel expression in microglia [151] and identify a new potential target in the treatment of neurodegenerative diseases. The in vivo role of these channels in AD is less known. However, given the central role of microglia in AD and the involvement of sodium channels in microglial activity, future in vivo research is needed to assess the therapeutic potential of sodium channels for AD.

## 6. Other Microglial Channels

Microglial voltage-gated proton channels (Hv1) become active in situations when there is an increased synthesis of nitrogen oxide (NOx) and ROS, thereby maintaining a stable intracellular pH level [152,153]. Due to the nature of these channels, pathologies that change the pH of the environment can benefit from Hv1 channel modulation. As pH is heavily impacted in ischemic lesions, it is not surprising that blocking Hv1 channels proved being beneficial. After limiting the blood flow to the brain of Hv1 knockout mice, an attenuated disruption of white matter integrity was observed. The same animals also had a decrease in the production of ROS and proinflammatory cytokines. Moreover, Hv1-/- mice showed increased oligodendrocyte precursor cell (OPC) proliferation and differentiation into oligodendrocytes and a dominant M2 microglial polarization, all of which suggest that Hv1 might be a therapeutic target [154].

As the link between hypoxia and AD is becoming more evident, the involvement of microglial Hv1 in cerebral pathologies is taking a center stage [152,155]. In AD, there is an increase of microglia ROS and as such, the function of these channels could be altered in AD, but a direct link between microglia Hv1 channels and AD has yet to be established. However, an in vitro study conducted on OPC co-cultured with either wild-type or Hv1-knockout microglia, showed that Hv1 deficiency decreases the production of proinflammatory mediators, an attenuation of OPC apoptosis and an increase in OPCs proliferation and differentiation [156]. Although the findings that imply an involvement of Hv1 channels in AD are compelling, in situ studies were unable to detect any proton conduction [157].

**Figure 2:**
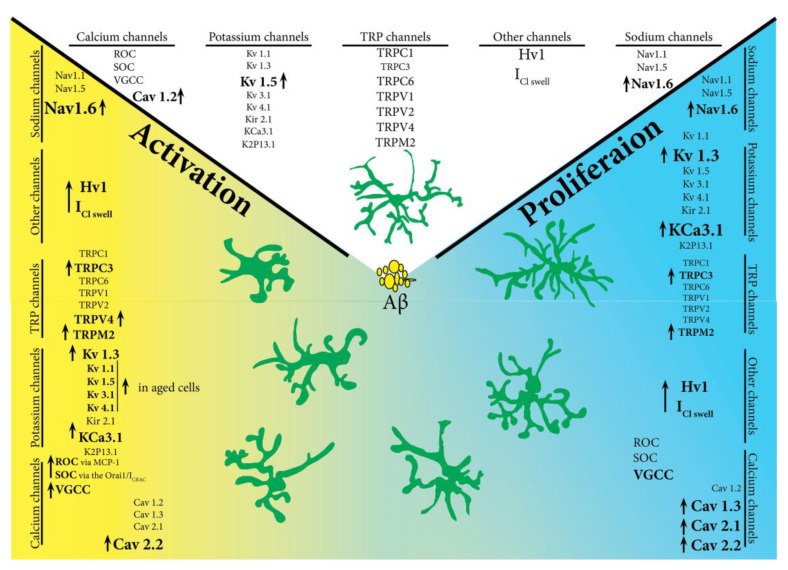
Changes in ion channel expression in the presence of amyloid beta.

**Microglia chloride channels.** Although no direct data links AD to microglia chloride channels, the expression of these channels increases regularly in response to neurotoxicity [158], possible because these channels are volume regulated anion channel, which are glutamate permeable [159]. Considering the involvement of the glutamatergic system in learning and memory, it is possible that a disturbance in glutamate neurotransmission can be one of the underlying causes of AD. Microglial activation, as seen in AD (Figure 2), generates massive release of glutamate, especially under acute neuropathology, leading to an increase in glutamate-induced chloride current causing neuronal damage [160,161]. The glutamate imbalance hypothesis was tested in a clinical trial that used memantine to regulate glutamate levels in patients with moderate and severe AD. The results, nevertheless, did not show any delay or halt in disease progression [162,163].

## 7. Ion Channels Modulation in Neurodegenerative Diseases. In Vivo Studies

Few studies targeting in vivo microglia ion channel modulation have been performed. This is mainly due to the lack of microglia specific ion channel blockers. Most of the in vivo studies aimed at blocking certain ion channels have shown a possible beneficial effect in the evolution of the disease. However, it was difficult to assess if the effect was a direct consequence of microglia channels modulation or other mechanisms were involved.

Microglia morphology can change in response to neuroinflammation and it has been taken for granted that any alteration in microglia response can be seen as a marker of successful ion channel modulation. This is especially true for potassium channels. For example, the extent of microglia activation did not change in response to blockade of ATP-mediated potassium channel, although, some improvements were observed in neurological deficits after middle cerebral artery occlusion (MCAo) [164].

When microglia detect “find me” or “eat me” signals, a plethora of intracellular changes occur. Some of them have a direct implication on ion channels, notable KCa3.1 and Kv1.3. Thus, using senicapoc to block KCa3.1 channels, improved the locomotor function in a mouse model of PD [48] while a decrease in neuroinflammation and degradation of neuronal synapses were reported in AD mice [47].

A cellular hallmark of microglia activation is the upregulation of Kv1.3 channels [39]. Blocking Kv1.3 with PAP-1A at 12 h after reperfusion, caused a reduction in microglial activation and perilesional inflammation accompanied by a decrease in the infarction area and neurological deficit in both adult male C57BL/6J mouse and adult male Wistar rat models of stroke [51]. However, not all studies were able to show a positive effect. For example, microglia activation was reported despite treatment with the K channels blocker, glibenclamide [164].

Due to the fact that the majority of so called “resting” microglia show almost no Ca signaling, it was hypothesized that blocking Ca signals in activated microglia could impact the outcome of some neuropathologies. This approach showed promise when the neuroprotective effect of Ca blockade was demonstrated in 16-week old male C57/B16 mice in which stroke was induced by MCAo. Thus, a reduction in the infarction area was observed in mice that were administered intra-arterial verapamil immediately after reperfusion. However, the results also indicated a decrease in astrogliosis and cellular apoptosis and an increase in neuron survival and functional outcome at 7 days after stroke [165]. Such effects cannot be attributed to microglia Ca blocking. The use of Verapamil in a mouse model of amyotrophic lateral sclerosis had a neuroprotective effect and increased the lifespan of mice most likely via a reduction in glia activation [166].

Ca blockers were also tested in clinical trials. One such trial was a pilot study in children showing that the administration of verapamil in addition to basic antiepileptic treatment reduced the incidence of all types of epileptic seizures in patients with Dravet syndrome but had no effect in patients with Lennox-Gastaut [167]. In another pilot study, performed on adults with epilepsy of different types and etiologies who were under antiepileptic treatment, co-administration of a small dose of 20 mg verapamil three times a day resulted in a drop in the frequency of epileptic in most patients. However, some of the participants showed no improvements [168]. Similarly, amlodipine (a non-selective calcium blocker) was also tested in different pathologies. In one report, the oral administration of amplodipine modulated superoxides production, improved neurological deficit, and reduced infarction area [70].

In vivo studies using Ca blockers have yielded conflicting results. Thus, treatment with isradipine, a Ca1.2 channel blocker, led to decreased levels of Aβ1-40 and 1-42 plaques [66] and prevented memory loss [65]. Nivaldipine, a non-selective calcium blocker, showed positive results for the cognitive function in a mouse model of AD [64]. However, the administration of nimodipine did not have the expected results in reducing Aβ1-42 secretion, suggesting a possible mechanism of amyloid plaque formation that is independent of Ca^2+^ channels [67]. One clinical trial using Ca blockers to treat AD patients, could not reach conclusive results, i.e., there were no significant differences between treated group and placebo. However, some positive effects were observed in several subgroups [68].

Besides the known neurological effects of sodium channels blockers, their administration to EAE mice diminished the number of activated microglia [142].

## 8. The Future of Microglia Ion Channels in Pathology

With our increased understanding of physiological and pathological roles ion channels play in the CNS, and with our interest shifting from a neuro-centric view of the CNS to a more integrative one, the role of ion channels in microglia are slowly starting to be explored in more detail.

Current studies conducted to investigate such roles are mainly performed on cultured cells and animal models. The biggest impediment in studying microglial ion channels is the species heterogeneity that exists between different microglia and the variability of channel expression during development and aging [169]. The current trend is to study in vitro, the interaction between microglia and human-induced pluripotent stem cells, especially those obtained from patients with specific neurodegenerative disorders, but the major disadvantage of these cultures is the lack of standard protocols for cell culture and differentiation [170]. For this reason, studies involving human subjects have not been successful [68].

We now know that the modulation of different channels can be achieved using pharmacological drugs such as flecainide [143], safinamide [143], carbamazepine [6], or phenytoin [6]. The neuroprotective effect of these drugs is mediated by blocking intracellular calcium accumulation in glial cells in response to neuroinflammation [171]. Indeed, recent studies showed that blocking calcium channels in microglia limits the pathological damage, thus paving the way for future therapeutic regimens in neurodegenerative diseases [172]. More specific effects, like inhibition of microglial phagocytic activity were seen by blocking KCa3.1 or Kv1.3 [173]. However, this seems to be just the tip of the iceberg as more and more studies have linked ion changes in microglia membrane to even more complex phenomena such as autophagy [174,175,176], cell death and apoptosis [177,178,179,180,181]. It seems that autophagy modulation by blocking Ca and KCa3.1 or Kv1.3 channels is a potential challenging issue that we need to address in aging and/or neurodegeneration.

## 9. Conclusions

The importance of ion balance in the normal life of a cell is well known. The specific problem of targeting the ion changes in pathology is not the lack of evidence shoving its importance, but rather the technical difficulties that needs to be overcome. This is especially true when one considers the impact of several studies that have tried to modulate microglia ion permeability by targeting the P2X and/or P2Y APT receptor families. Multiple studies have shown a benefit in blocking microglia P2X channels after epileptic seizures [182,183], stroke [184], and traumatic brain injury [185]. The main problem in the field is the lack of results produced in in vivo studies that target microglia specific ion channels. A better understanding of the molecular pathways underlying ion channels activity in microglia shall allow us to develop more specific microglia channel blockers for the treatment of CNS pathologies.

## Data Availability

All data can be obtained upon request.
